# Immunoexpression of LGR4 and Β-Catenin in Gastric Cancer and Normal Gastric Mucosa

**DOI:** 10.31557/APJCP.2019.20.2.519

**Published:** 2019

**Authors:** Susana Moreira de Souza, Adriana Estela Flores Valiente, Kélvia Miranda Sá, Camila de Carvalho Juanes, Bruno Jucá Rodrigues, Amanda Cristina Crispim Farias, Caroline Cavalcante Campelo, Paulo Goberlânio de Barros Silva, Paulo Roberto Carvalho de Almeida

**Affiliations:** 1 *Department of Pathology and Forensic Medicine, Faculty of Medicine,*; 3 *Postgraduate Program in Dentistry, Department of Dental Clinic, Federal University of Ceará,*; 2 *Fortaleza Air Base, Aeronautics, Armed Forces, Brazil.*

**Keywords:** Gastric cancer, stem cell, LGR4, β-catenin

## Abstract

**Background:**

We evaluated the immunoexpression of LGR4 and β-catenin in primary gastric carcinomas, lymph node metastases and histologically normal gastric mucosa in the surgical margins of gastric primary tumours.

**Methods:**

We performed a cross-sectional, observational study, based on 75 gastric carcinoma specimens from gastrectomies conducted at the hospital of the Federal University of Ceará, Brazil. The samples were analysed by tissue microarray and immunohistochemistry. Chi-square, Fisher’s exact test and Pearson’s linear regression were used in this study.

**Results:**

LGR4 expression was greater in the histologically normal gastric mucosa (basal third of the epithelial thickness) of the tumour surgical resection margin than in the cases of primary carcinomas (P<0.001, mainly diffuse-histotype cancer margins), and also in the number of cells stained in the normal mucosa (P<0.0001). Primary intestinal-type carcinomas showed greater positivity for LGR4 than diffuse tumours (59% vs 13%, P<0.0001) and in these the positivity was higher in the metastases (P=0.0242). The membranous immunoexpression of β-catenin was ubiquitous in the normal mucosa and present in 2/3 of the positive carcinomas. In only one case, nuclear β-catenin expression was observed. Most LGR4-positive cases were stained for membranous β-catenin but not the opposite (P<0.01).

**Conclusion:**

LGR4 is a likely biomarker of stem cells in the normal gastric mucosa and carcinomas of the stomach, not specific to cancer cells and positively associated with cell proliferation. LGR4 immunoexpression is more frequent and found in a larger number of cells in normal tissues than in tumour samples. Expression of β-catenin in the junctional membrane-complex occurred predominantly, in positive cases of gastric carcinomas and very rarely in the nucleus. LGR4 apparently influenced the membranous expression of β-catenin. These findings suggest a controversial role for LGR4, related to proliferative status and inversely related to tumour progression, in contrast to most previous reports.

## Introduction

Gastric cancer is the fifth most common neoplasm worldwide, preceded by lung, breast, colorectal and prostate cancer. In mortality, it ranks third after lung and liver cancer (Ferlay et al., 2013). According to Lauren’s classification, there are two main types of adenocarcinomas, with distinct histological, clinical and epidemiological characteristics: the intestinal type and the diffuse type (Lauren, 1965).

Cancer stem cells are associated with tumour initiation, development and metastatic dissemination (Ishigami et al., 2010). Molecules expressed in normal stem cells can be used as biomarkers of cancer stem cells (Wang et al., 2011). New candidates for stem cell biomarkers have been investigated recently. Among these, the G protein receptors rich in leucine residues (LGRs) monitor important physiological and pathological functions (Barker and Clevers, 2010; Nakata et al., 2014). LGR4, a member of this family of receptors, participates in cell signalling, activating transcription factors related to tumour formation, potentiating the Wnt signalling pathway and playing an important role in the development of multiple organs and cancer progression (Wu et al., 2013; Xiao and Chen, 2015; Xu and Hou, 2017).

β-catenin is a perimembranous cytoplasmic protein that interacts with the cadherins of the intercellular junctional complex in the adhesion zones. When excess β-catenin is present in the cytoplasm, it can migrate to the nucleus where it acts as a transcription factor for cell proliferation. In addition, β-catenin participates in cellular communication, mainly related to the Wnt signalling pathway (Bourroul et al., 2016). Mutations, cytoplasmic/nuclear overexpression of β-catenin and their membrane loss or decrease have been associated with several types of neoplasms including colorectal carcinoma and gastric carcinoma (Clements et al., 2002).

In gastric cancer, LGR4 is a potential stem cell marker, and may be involved in the activation of β-catenin similar to LGR5 and LGR6 (Carmon et al., 2011; Steffen et al., 2012; Ruffner et al., 2012). Moreover, in cell signalling pathways, it activates transcription factors related to tumour formation (Ruffner et al., 2012; Wu et al., 2013), but these aspects are not yet clear. The aim of this study is to evaluate the immunoexpression of LGR4 and β-catenin in primary gastric carcinomas in both histotypes, lymph node metastases and histologically normal gastric mucosa at the tumour resection margins and to correlate the expression of these potential biomarkers with clinical variables such as age, sex and tumour staging.

## Materials and Methods

*Patients and samples*

The study is a cross-sectional observational study of 75 paraffin-embedded gastrectomy specimens collected at the Department of Pathology and Legal Medicine of the Federal University of Ceará (DPML / UFC). Tissue samples of primary tumours (n=75), lymph node metastases (n=26) and histologically normal gastric mucosa at the tumour surgical margin (n=60) were selected, fixed in 10% formalin, processed and embedded in paraffin and then evaluated in histological sections stained with haematoxylin-eosin. In the evaluation of β-catenin, four cases of primary tumours and six lymph node metastases were not appropriate for analysis and were excluded. Similarly, eight cases of histologically normal mucosa were not suitable for LGR4 analysis and were also excluded. Specimens with inadequate fixation, hemorrhage and necrosis were not included in the original sampling.

The cases were classified according to Lauren’s classification into two main types of adenocarcinomas, with distinct histological, clinical and epidemiological characteristics. The intestinal type, which is composed of cohesive neoplastic cells that form tubular structures that resemble the glands and the diffuse type, consisting of isolated cells that are dispersed throughout the stomach wall (Lauren, 1965; Lauwers et al., 2010).

A tissue microarray (TMA) technique was used in this study (Kononen et al, 1998). Immunohistochemistry was performed using a Dako™ platform and Abcam™primary monoclonal antibodies anti-LGR4 (GR125979-19) and anti-β-catenin (GR205416-1) both diluted 1/500.

As external positive controls for LGR4, normal bowel tissue (Yamanoi et al., 2013) and normal kidney tissue (Simon et al., 2012) were utilized. For β-catenin, an intestine sample was used as positive control (Jawhari et al., 1997). For the negative control, immunohistochemistry on two samples was performed in the absence of primary antibody.

*Evaluation of immunostaining*

LGR4: based on previous studies, at least one cell with membranous or cytoplasmic labeling is required for the sample to be considered positive (Ghaffarzadehgan et al., 2008; Choi et al., 2009; Ishigami et al., 2010). Counts of stained cells (direct visual counting by groups of five cells) from 100 to 1,000 cells/case using an appropriate optical system (Carl Zeiss™) were performed. The fields were counted sequentially from a random point (Hashimoto et al., 2014) at 400× magnification, until reaching the number of cells in the last field evaluated. Membranous or nuclear β-catenin expression was considered positive when the number of stained cells was ≥ 10% (Nagy et al., 2017).

Histological preparations for LGR4 and β-catenin immunostaining were double-blind investigated by different researchers (Souza, SM and Almeida, PR). When the results for the same sample were different, a third investigation was performed for confirmation.

*Statistical analysis*

The relationship between the differential expression of LGR4 and β-catenin and the clinical and pathological features was assessed using Fisher’s exact test or chi-square test, with a P value <0.05 considered statistically significant. The numbers of stained and unstained cells from the LGR4 positive cases of tumours and normal mucosa were analysed by the Kolmogorov-Smirnov normality test (P>0.05). The positive cell counts of the two researchers were compared by Pearson’s correlation, with a very strong (r=0.905) statistically significant correlation coefficient (P<0.001) validating its calibration. GraphPad Prism 6™ software was used for statistical analysis and plotting.

*Ethical Aspects*

This project was approved by the Ethical Committee of the Federal University of Ceará, Fortaleza, CE, Brazil (Process number 2004326).

## Results

**Figure1:**
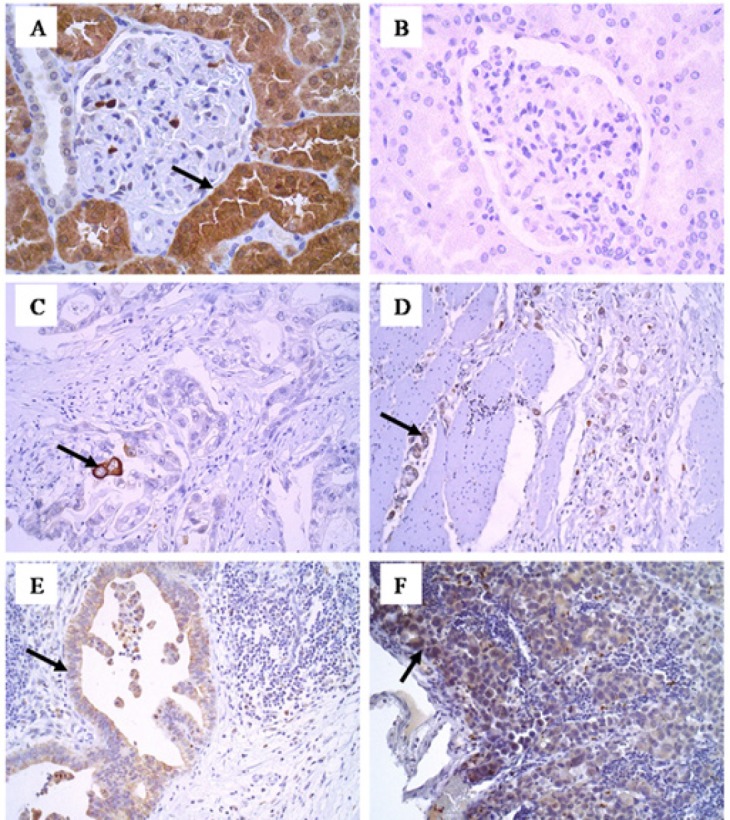
Membranous and Cytoplasmic Immunoexpression of LGR4. (A) Positive control, cytoplasmic staining in the renal tubular cells (400x). (B) Negative control (400x). (C) Staining in rare cells of the primary intestinal tumour (200x). (D)Positivity in diffuse primary tumour cells (200x). (E) Expression in lymph node metastasis of intestinal tumour cells (200x). (F) Positivity in lymph node metastasis of diffuse type tumour cells (200x). (G) Intense expression in cells of histologically normal mucosa (200x)

The general clinicopathological variables data and the LGR4 and β-catenin immunoexpression obtained in this study are shown in table 1 and in figures 1 and 4.

**Table 1:** Immunoexpression of LGR4 and β-Catenin in Primary Tumours and the Clinicopathological Variables

Clinicopathological Variables										
	LGR4					β-catenin				
	n	%	+	-	P	n	%	+	-	P
Sex										
Male	44	59	17 (39%)	27 (61%)	0.6310	41	58	28 (68%)	13 (32%)	0.6155
Female	31	41	10 (32%)	21 (68%)		30	42	18 (60%)	12 (40%)	
Total	75					71				
Age										
<50	08	11	02 (25%)	06 (75%)	0.7031	08	11	07 (88%)	01 (12%)	0.2455
≥ 50	67	89	25 (37%)	42 (63%)		63	89	39 (62%)	24 (38%)	
Total	75					71				
Histological type										
Intestinal	37	49	22 (59%)	15 (41%)	0.0001*	36	51	27 (75%)	09 (25%)	0.085
Diffuse	38	51	05 (13%)	33 (87%)		35	49	19 (54%)	16 (46%)	
Total	75					71				
Location										
Body	44	59	14 (32%)	30 (68%)	0.7148	40	56	23 (58%)	17 (42%)	0.4054
Antrum	19	25	07 (37%)	12 (63%)		19	27	15 (79%)	04 (21%)	
Body + Antrum	10	13	05 (50%)	05 (50%)		10	14	07 (70%)	03 (30%)	
Whole stomach	02	03	01 (50%)	01 (50%)		02	03	01 (50%)	01 (50%)	
Total	75					71				
Dimension of the tumor										
<5cm	19	25	08 (42%)	11 (58%)	0.5852	18	25	13 (72%)	05 (28%)	0.5719
≥ 5cm	56	75	19 (34%)	37 (66%)		53	75	33 (62%)	20 (38%)	
Total	75					71				
Angiolymphatic invasion										
Absent	38	51	17 (45%)	21 (55%)	0.1498	38	54	29 (76%)	09 (24%)	0.0456*
Present	37	49	10 (27%)	27 (73%)		33	46	17 (52%)	16 (48%)	
Total	75					71				
Perineural invasion										
Absent	55	73	24 (44%)	31 (56%)	0.0294*	52	73	38 (73%)	14 (27%)	0.0242*
Present	20	27	03 (15%)	17 (85%)		19	27	08 (42%)	11 (58%)	
Total	75					71				
Degree of invasion										
T1	11	15	07 (64%)	04 (36%)	0.0489*	11	15	7 (64%)	04 (36%)	1.0000
T2-T4	64	85	20 (31%)	44 (69%)		60	85	39 (65%)	21 (35%)	
Total	75					71				
Lymph node metastasis										
N0	22	29	13 (59%)	09 (41%)	0.0095*	21	30	17 (81%)	04 (19%)	0.1015
N1-N3	53	71	14 (26%)	39 (74%)		50	70	29 (58%)	21 (42%)	
Total	75					71				

*P<0.05, Fisher exact test; **4 samples were not appropriated for analyses.

**Table 2:** Immunoexpression of LGR4 and β-catenin in Primary Tumours, Lymph Node Metastases and Histologically Normal Mucosa of Patients with Intestinal and Diffuse Gastric Carcinoma

Histological type	LGR4					β-catenin				
	n	%	+	-	*P*	n	%	+	-	*P*
Primary tumor										
Intestinal	37	49	22 (59%)	15 (41%)	0.0001*	36	51	27 (75%)	09 (25%)	0.0850
Diffuse	38	51	05 (13%)	33 (87%)		35	49	19 (54%)	16 (46%)	
Total	75		27 (36%)	48 (64%)		71		46 (65%)	25 (35%)	
Lymph node metastasis										
Intestinal	11	42	04 (36%)	07 (64%)	0.7007	08	40	06 (75%)	02 (25%)	0.1968
Diffuse	15	58	07 (47%)	08 (53%)		12	60	05 (42%)	07 (58%)	
Total	26		11 (42%)	15 (58%)		20		11 (55%	09 (45%)	
Histologically normal mucosa**										
Intestinal	28	54	27 (96%)	01 (04%)	1.0000	32	53	28 (88%)	04 (12%)	0.6754
Diffuse	24	46	23 (96%)	01 (04%)		28	47	26 (93%)	02 (07%)	
Total	52		50 (96%	02 (04%)		60		54 (90%)	06 (10%)	

*, P<0.05, Fisher exact test; **, Surgical margin of tumour.

**Table 3:** Expression of LGR4 in Primary Tumours, Lymph Node Metastases and Histologically Normal Mucosa with Gastric Carcinoma Histotype (Intestinal and Diffuse) by Cell Count

	Immunoexpression of LGR4			
	n	+	-	*P*
Histological type Primary tumor				
Intestinal	21,001	7,321 (35%)	13,680 (65%)	0.0001*
Diffuse	3,969	990 (25%)	2,979 (75%)	
Total	24,970	8,311 (33%)	16,659 (67%)	
Lymph node metastasis				
Intestinal	7,320	3,142 (43%)	4,178 (57%)	0.0018*
Diffuse	1,676	649 (39%)	1,027 (61%)	
Total	8,996	3,791 (42%)	5,205 (58%)	
Histologically normal mucosa*				
Intestinal	18,147	6,457 (36%)	11,690 (64%)	0.0001*
Diffuse	15,407	7,024 (46%)	8,383 (54%)	
Total	33,554	13,481 (40%)	20,073 (60%)	

*, P<0.05, Fisher exact test and Qui-square; **, Surgical margin of tumour.

**Figure 2:**
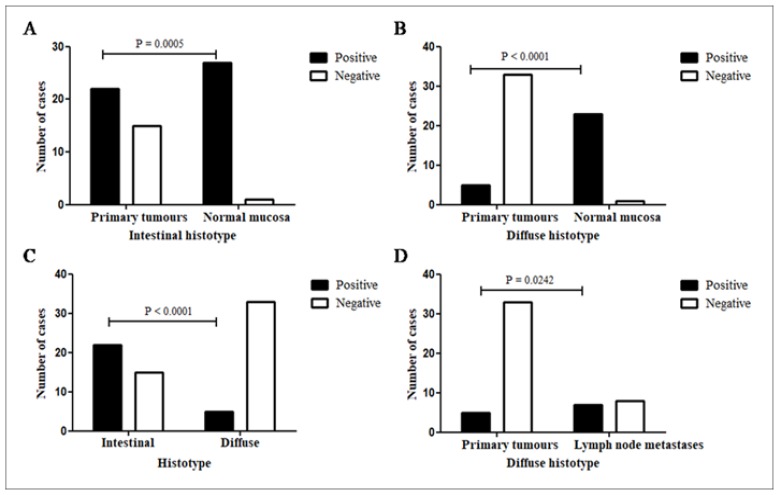
LGR4 Immunoexpression. (A), Primary gastric carcinoma of intestinal histotype vs histologically normal gastric mucosa at the surgical margin of the same histotype; (B), Diffuse primary gastric carcinoma vs histologically normal gastric mucosa at the surgical margin of the same histotype; (C), Primary gastric cancer according histotypes: intestinal and diffuse. (D) Diffuse primary gastric cancer vs diffuse lymph node metastasis. P <0.05. Fisher exact test

**Figure 3:**
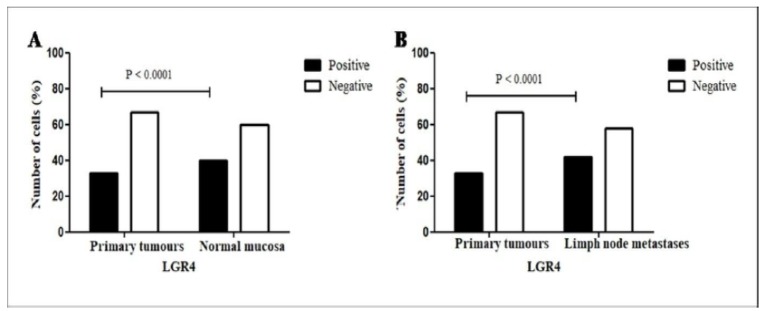
Positive Expression by Cell Counts for LGR4. (A) Primary tumour vs normal gastric mucosa. (B) Primary tumour vs lymph node metastasis. P <0.05. Chi-square

**Figure 4:**
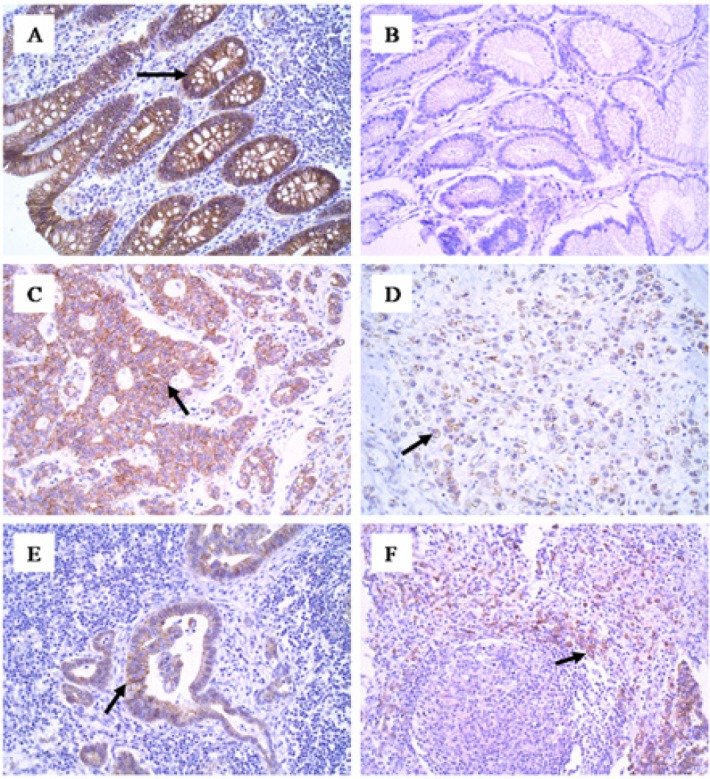
β-catenin Membranous Immunoexpression. (A), Positive control, membrane staining in intestinal mucosal epithelial cells (200x); (B), Negative control (200x); (C), Staining of epithelial cells, gastric cancer of the intestinal histotype (200x); (D), Positivity in epithelial cells, gastric cancer of the diffuse histotype (200x); (E), Expression in metastatic cells of gastric cancer of the intestinal type in the lymph node (200x); (F), Staining in metastatic cells of gastric cancer of the diffuse type in the lymph node (200x); (G), Membrane positivity in epithelial cells of the histologically normal mucosa (200x).

**Figure 5:**
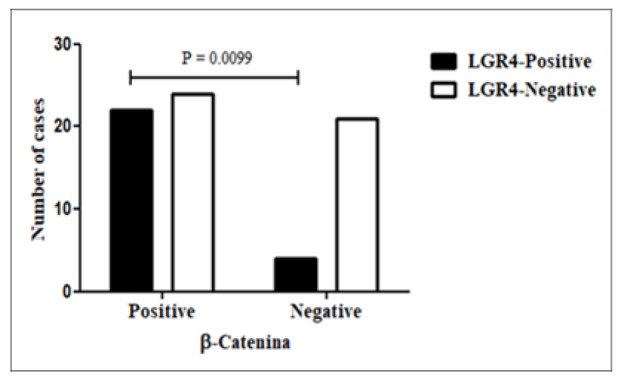
β-catenin Immunoexpression. (A) Primary gastric carcinomas vs histologically normal gastric mucosa at the surgical resection margin. (B) In diffuse primary gastric cancer vs histologically normal gastric mucosa at the surgical margin. P<0.05. Fisher exact test.

**Figure 6:**
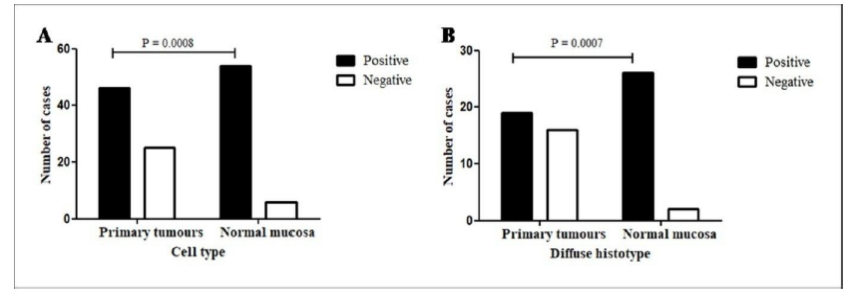
Comparison of Positive Immunoexpression of LGR4 and β-catenin in the Primary Tumour, P=0.0099. Fisher Exact Test

*Distribution of LGR4 immunoexpression in the normal gastric mucosa, primary gastric carcinomas and lymph node metastases*

The expression of LGR4 in the histologically normal mucosa in a total of 50 out of 52 cases (96%) was higher than in the primary tumours (27/75=36%, without distinguishing between histotypes), with a significant difference (P<0.0001), as shown in table 2. A higher positivity was seen in the histologically normal mucosa at the surgical margin compared to tumour cells in the intestinal histotype of the primary tumours (27/28=96% and 22/37=59%, respectively; P=0.0005; Figure 2A). The same was true for the diffuse histotype, with a much higher positivity in the histologically normal mucosa (23/24=96%) than in the diffuse primary tumour (5/38=13%; P<0.0001; Figure 2B). Comparing the LGR4 expression in the primary carcinomas, a greater positivity is observed in the intestinal tumours (22/37=59%) than in diffuse type (05/38=13%; P<0.0001; Table 2; Figure 2C). Diffuse histotype positivity was much higher in metastases (primary tumour: 5/38=13%; lymph node: 7/15=47%; P=0.0242, figure 2D) but the same not occurred comparing intestinal histotype in both sites (Table 2). Other comparisons did not differ significantly.

*Immunoexpression of LGR4 in the histologically normal mucosa, primary carcinomas and lymph node metastases, based on positive cell counts*

The counts of LGR4-positive cells in the primary tumours, lymph node metastases and normal mucosa were evaluated, as shown in Table 3. Greater expression in histologically normal mucosa cells (13481/33554=40%) was observed in relation to the whole group of primary tumours (8311/24970=33%, P<0.0001; Figure 3A).

The immunoexpression for LGR4 in the primary tumour in relation to the number of positive cells was significantly higher in the intestinal type (7321/21001=35%) than in the diffuse type (990/3969=25%; P<0.001). In the histologically normal mucosa, at the margins of the diffuse subtype, the immunostaining was significantly higher (7024/15407=46%) than in the intestinal type (6457/18147=36%, P<0.0001). When evaluating the total cells immunostained in the metastases (3791/8996=42%), it was observed that it was well above the total in primary carcinomas (8311/24970=33%, P<0.0001; Figure 3B). In the lymph node, cell positivity in intestinal histotype metastases was more frequent (3142/7320=43%) than that in the diffuse types (649/1676=39%, P=0.0018).

*β-catenin immunoexpression and clinicopathological variables*

β-catenin immunoexpression was observed in the cell membrane/cytoplasm in all normal mucosa cases and in all carcinoma positive cases (Figure 4), except for one case of metastatic intestinal-type tumour, which this protein was immune labelled in the cell nucleus. With regard to β-catenin membranous immunoexpression and its clinical-pathological variables (Table 1), there was a predominance of positivity in the absence of angiolymphatic invasion (29/38=76%) compared to its presence (17/33=52%, P=0.0456,). The positivity was higher in the absence of perineural invasion (38/52=73%) than to its presence (8/19=42%, P=0.0242).

*Distribution of β-catenin immunoexpression in histologically normal gastric mucosa, primary gastric carcinomas and lymph node metastasis*

Considering all the analysed samples, the positive expression of β-catenin in the histologically normal mucosa (54/60=90%) was greater than that in the primary tumours without distinguishing between histotypes (46/71=65%, P=0.0008, Table 2; Figure 5A).

Regarding the diffuse histotype, a higher positivity in the histologically normal mucosa (26/28=93%) was observed compared to that in the primary tumour (19/35=54%) (P=0.0007, Table 2; Figure 5B).

No significant difference was seen comparing primary intestinal tumours with respective surgical margins, histotypes at each site, or each histotype in the stomach and lymph node.

*Comparison of positive immunoexpression of LGR4 and β-catenin*

The majority of LGR4 positive cases (both histotypes altogether) were also β-catenin positive (22/26=85%), but the number of β-catenin positive cases that were also LGR4 positive was only 48% (22/46; P=0.0099; Figure 6, data not previously shown). The differences for each histotype were not statistically significant.

## Discussion

In this study, the LGR4 membranous/cytoplasmic expression was present in almost all cases (96%) in the histologically normal gastric mucosa, always in the basal third of the epithelial thickness. This site is involved with the cellular proliferation of the mucosal renewal, where cells are less differentiated as stem cells and progenitor cells. At the same time, higher LGR4 positivity was observed in the normal mucosa than in the tumour tissue, with a highly significant difference (93% and 36%, P<0.0001). These findings are indicative of LGR4 being involved in cell proliferation, whether of normal or tumour cells, without distinguishing them. LGR4 possibly represents a marker of stem/progenitor cells lato sensu, not being characteristic or specific of neoplastic cells.

A few reports have compared the presence of LGR4 in normal and tumour mucosa. Wu et al., (2013) found significantly higher LGR4 expression in tumour cells than in normal tissues in histological sections of large intestinal mucosa and of colonic tumours. This finding is consistent with our data regarding the presence of this marker in the two types of tissue but discordant regarding the higher frequency in their cancer samples.

Some recent studies have investigated LGR4 in stomach cancer. Steffen et al., (2012) observed a correlation between LGR4 expression and lymph node dissemination in human carcinoma samples. In human colorectal carcinomas, Wu et al., (2013 ) reported that LGR4 is a poor prognostic factor for increasing tumour invasion and metastasis when it is associated with an increase of nuclear β-catenin via Wnt. In an in vitro study with a culture of various types of cancer cells (stomach, liver, prostate and breast), Zhu et al., (2013) also observed that LGR4 overexpression was associated with increased expression of nuclear β-catenin. In addition, another in vitro and experimental study (Yue et al., 2018) reported that LGR4 was associated with a poor prognosis because its reduction decreased the number of cancer stem cells and of cells in epithelial-mesenchymal transition. All these reports point to a role of the pro-carcinogenic LGR4 in promoting tumour progression.

Although in this study LGR4 was present in tumour samples, its expression was more common in those cases without lymph node metastases, with less local invasion and in the absence of perineural invasion, i.e., associated with clinical variables of better prognosis. Therefore, its expression was inversely related to tumour progression, the opposite of what has been generally reported (Steffen et al., 2012; Wu et al., 2013; Zhu et al., 2013; Yue et al., 2018).

The cell count in the positive cases of LGR4 strengthens the information stated previously regarding the comparison between the number of normal and tumour positive cases: a much higher percentage of stained cells in the normal mucosa compared to the percentage of positive tumour cells. Among these cases, cell staining in primary gastric tumours was greater than that of the respective lymph node metastases. Moreover, in primary carcinomas of the intestinal histotype, the LGR4 immunoexpression was significantly higher than that of the diffuse type , a lesser differentiated histotype , finding consistent with that of Steffen et al., ( 2012). These findings suggest that LGR4 is a valuable biomarker of cells linked to proliferation (stem cells/progenitors) in normal and cancer contexts, but that it plays a role in opposing tumour progression.

In recent studies, LGR4 has been shown to promote the proliferation of cancer cells through the activation of the Wnt/β-catenin signalling pathway that plays important roles in stem cell biology, tissue homeostasis and cancer (Carmon et al., 2011; Glinka et al., 2011; Ruffner et al., 2012; Zhu et al., 2013). The key mediator of this signalling pathway is β-catenin that is found dynamically at multiple subcellular locations, including intercellular junctions, cytoplasm and nucleus (Reya and Clevers, 2005). Mutations in β-catenin may cause activation of the Wnt pathway in gastric cancer (Clements et al., 2002), when they favor the nuclear localization of this protein. The cytoplasmic and/or nuclear localization of β-catenin can be used to estimate Wnt pathway activity. These relationships between LGR and β-catenin proteins during carcinogenesis and possibly during tumour progression prompted us to study this protein and LGR4 in the same samples of normal and tumour gastric mucosa. In this study, it was observed that immunostaining for β-catenin was universally present at membrane localization in histologically normal mucosal cells as expected. In the tumour samples, membranous immunostaining was present in 2/3 of the cases. And in only one case, there was nuclear staining in the lymph node metastasis whose primary tumour was of the intestinal histotype. This result was similar to that of Lins et al., (2016) who found only two cases (2/64=3.1%) of nuclear positivity in intestinal gastric cancer samples. The rarity of the nuclear expression of β-catenin in our case series drastically reduces the importance of the Wnt pathway during the carcinogenesis of these cases. However, Wang et al., (2015) found nuclear positivity in 31.6% of their cases, with predominance in the intestinal histotype. In a different way, Wu et al. (2013) observed high β-catenin expression in 52.9% of the colorectal cancer (CRC) cases, involving the cytoplasm (70.6%) and nucleus (26.5%) of the cells.

The evaluation of β-catenin expression in relation to clinicopathological variables in this study revealed a predominance of positivity (membranous expression) in the absence of angiolymphatic and perineural invasion. These findings are consistent with the suppressor role of β-catenin during tumour progression, when localized in the junctional membrane complex, favouring adhesion between cells.

Could there be a possible cause and effect association between LGR4 and β-catenin expression or vice versa based on the results of this study? When comparing the immunoexpression of LGR4 and β-catenin membranes, it was observed that when LGR4 was positive, the great majority of β-catenin (85%) was also positive. However, when β-catenin was positive, only half the cases stained by LGR4 were positive, the difference between the two comparisons being highly significant (P<0.01). These data suggest that LGR4 may influence the expression of β-catenin in the cell membrane but that the expression of the latter does not appear to significantly influence the positivity of LGR4. This observation suggests a possible mechanism of anti-neoplastic action of LGR4. No previous studies were found for comparison.

In summary, LGR4 is a potential biomarker of stem cells in normal gastric mucosa and carcinomas of the stomach, not specific to cancer cells and positively associated with cell proliferation. Its immunoexpression is more frequent and occurs in a larger number of cells in normal tissues than in tumour samples. Expression of β-catenin in the junctional membrane complex occurred predominantly in positive cases of gastric carcinomas, and immunostaining of this protein in the nucleus was extremely rare. LGR4 apparently influenced β-catenin membranous expression but not the opposite. The findings from this study suggest a controversial role for LGR4, related to proliferative status and inversely related to tumour progression, in contrast to most previous reports.

## Funding Statement

This project was partially funded from the Public Call MCTI/CNPq Nº 14/2013 - Universal/Universal, process Nº 483318/2013-2.

## Statement conflict of Interest

The authors state that they have no conflicts of interest.
